# Understanding and leveraging phenotypic plasticity during metastasis formation

**DOI:** 10.1038/s41540-023-00309-1

**Published:** 2023-10-06

**Authors:** Saumil Shah, Lisa-Marie Philipp, Stefano Giaimo, Susanne Sebens, Arne Traulsen, Michael Raatz

**Affiliations:** 1 Department of Theoretical Biology, Max Planck Institute for Evolutionary Biology, August-Thienemann-Str. 2, 24306 Plön, Germany; 2 Institute for Experimental Cancer Research, Kiel University and University Hospital Schleswig-Holstein, Campus Kiel, Arnold-Heller-Str. 3, Building U30, Entrance 1, 24105 Kiel, Germany

**Keywords:** Cancer, Dynamical systems, Population dynamics

## Abstract

Cancer metastasis is the process of detrimental systemic spread and the primary cause of cancer-related fatalities. Successful metastasis formation requires tumor cells to be proliferative and invasive; however, cells cannot be effective at both tasks simultaneously. Tumor cells compensate for this trade-off by changing their phenotype during metastasis formation through phenotypic plasticity. Given the changing selection pressures and competitive interactions that tumor cells face, it is poorly understood how plasticity shapes the process of metastasis formation. Here, we develop an ecology-inspired mathematical model with phenotypic plasticity and resource competition between phenotypes to address this knowledge gap. We find that phenotypically plastic tumor cell populations attain a stable phenotype equilibrium that maintains tumor cell heterogeneity. Considering treatment types inspired by chemo- and immunotherapy, we highlight that plasticity can protect tumors against interventions. Turning this strength into a weakness, we corroborate current clinical practices to use plasticity as a target for adjuvant therapy. We present a parsimonious view of tumor plasticity-driven metastasis that is quantitative and experimentally testable, and thus potentially improving the mechanistic understanding of metastasis at the cell population level, and its treatment consequences.

## Introduction

Cancer metastasis formation, or the spread and growth of tumor cells throughout the body, is the cause of more than 60% of cancer-related deaths^1^. Despite many decades of drug development for cancer, the survival rate for patients with metastatic cancer remains low^2,3^. The process of metastasis is a multistage process that includes local invasion by the tumor cells, invading into the circulatory system, survival in circulation, arrest at a distant tissue, exiting the circulation, survival, adaptation and outgrowth in a new environment^4–6^. Tumor cells face selection for proliferation locally at each site of cancer, and for invasiveness and motility during the spread between two sites. These changing selection pressures during metastasis are driven by environmental factors and interactions with different surroundings^7,8^.

How tumor cells respond to these frequently changing environmental conditions is crucial for their persistence. Tumor cell populations possess several adaptive strategies to cope with such fluctuating environments. A subset of these strategies relies on diversity-generating mechanisms. In addition to genetic mechanisms, such as mutations, copy number alterations, and translocations, there are non-genetic mechanisms to generate diversity, such as epigenetic regulation, stochastic gene expression, and cellular differentiation hierarchies^7,9^. All these factors are internal to the cell. In addition, the effect of the local environment can be substantial; local variations in the tumor microenvironment may generate functional heterogeneity among clonal tumor cells^7,8^. An early review by West-Eberhard^10^ emphasizes plasticity as a diversity-generating mechanism and its contribution to altering traits. Plasticity also affects population and eco-evolutionary dynamics^11^.

One prominent outcome of epithelial tumor cell plasticity is the range of phenotypes and transitions on the spectrum from epithelium to mesenchyme. Epithelial cells are proliferative but cannot move, while mesenchymal cells are motile and invasive but proliferate slowly^12^. Both proliferative and invasive phenotypes are crucial for tumor progression and metastasis. Thus, shifts in the phenotype of tumor cells by epithelial-mesenchymal plasticity are salient features of metastatic cancers^13–16^. Consequently, during the epithelial-to-mesenchymal transition, partial mesenchymal features are gained, and epithelial features are progressively lost, leading to altered surface marker expression, decreased cell-cell adhesion, and motility-facilitating cytoskeleton reorganization^17^. The reverse change happens during the mesenchymal-to-epithelial transition. These transitions result in the presence of hybrid phenotypes along the continuous spectrum between epithelial and mesenchymal phenotypes. This continuous spectrum is often discretized into a number of hybrid phenotypes, with varying estimates on the number of these hybrid phenotypes^18–22^. The plasticity between epithelial and mesenchymal phenotypes was found to be promoted by interactions with the tumor microenvironment. Among others, high levels of Transforming Growth Factor *β* (TGF-*β*) is a critical driving factor toward more mesenchymal phenotypes^8,17,23^.

Different treatment types select differently on this plasticity^24^. The current first-line treatment for many cancers is chemotherapy which targets cell proliferation. A more recent treatment option for some cancers is immunotherapy which comprises antibody-based or cellular therapies targeting molecules present on the cell membrane of the cancer cells. These differential selection pressures suggest classifying treatments into growth-dependent or growth-independent treatments. As epithelial-like phenotypes proliferate faster, they are more sensitive to chemotherapy than mesenchymal phenotypes. Immunotherapy should target all phenotypes similarly, assuming that it does not target surface markers that change during epithelial-mesenchymal transition. Adjuvant therapies, such as TGF-*β* blockers, are promising candidates to modify the transition process and, thus, ultimately, the phenotypic plasticity of tumor cells^25–27^. As TGF-*β* promotes the mesenchymal phenotype^26,27^ and TGF-*β* blockers improve chemotherapy^25^, it is suspected that the gene regulatory network of the epithelial-mesenchymal transition could be a key to control the balance of phenotypes and thus the treatment outcome^28,29^. This gene regulatory network consists of cell fate-controlling signaling pathways like TGF-*β*, WNT, and NOTCH that mediate the recruitment of epithelial-mesenchymal-transition-inducing transcription factors ZEB, SNAIL, SLUG, TWIST, or miR-200^17,29–32^.

Mathematical modeling presents a valuable approach to understand the mechanisms and consequences of genetic and non-genetic diversity-generating mechanisms. Along this line Zhou et al.^33^, modeled the hierarchical cellular diversity created by differentiation and de-differentiation, with stem cells at the root of the hierarchy and specialized cells at its leaves. Gupta et al.^34^ and Li and Thirumalai^35^ showed with experiments and mathematical modeling that phenotypically plastic breast cancers approach a phenotypic equilibrium and can maintain phenotypic heterogeneity. A mathematical model of phenotype switching between drug-sensitive and drug-resistant cells in non-small cell lung cancer^36^ also shows a similar behavior. Such plastic mechanisms in tumor cells cause reduced efficacy of chemotherapy and acquired therapeutic resistance^13,37,38^. In a spatial, multi-organ model, Franssen and Chaplain^39^ focus specifically on epithelial-mesenchymal transitions during metastatic spread without treatment. In a recent paper, we modeled the phenotype switching between fast and slowly proliferating B cells during the relapse dynamics in acute lymphoblastic leukemia and investigated the impacts of treatment on phenotypic heterogeneity^24^.

In the present study, we propose an unconform yet parsimonious view of the phenotype transitions without explicitly considering the microenvironment. This view considers the trait variation present in a tumor population and consequently the heterogeneous response to drugs as an inherent property of the tumor. We also discuss feasible observables that could be measured in patient-derived tumors to test our hypothesis. In this study, we focus on (i) how different characteristics of plasticity shape the phenotype distribution, (ii) how this distribution determines the transition dynamics between epithelial and mesenchymal phenotypes at primary and secondary tumor sites, and (iii) how these dynamics are affected by chemo- or immunotherapy, and additionally by a transition-modulating therapy. With the proposal of a practical transition modulating therapy we emphasize the phenotypic plasticity as a viable clinical target.

## Results

### Overview

**Fig. 1:**
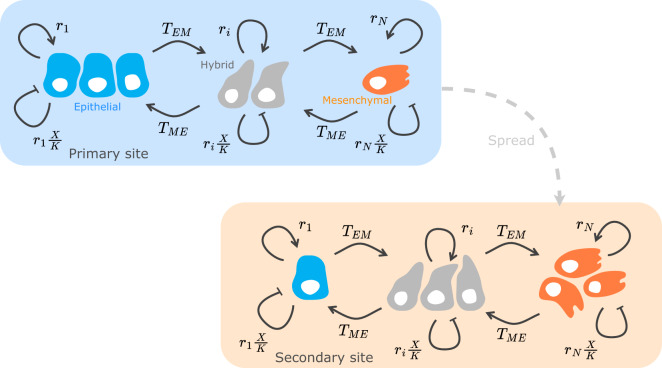
Model structure capturing phenotypic heterogeneity at the primary and secondary tumor site. We focus on the competitive growth of heterogeneous tumor cell populations at each tumor site. The dashed arrow represents spread of cancer cells between the primary and secondary sites. We do not model the spread between tumor sites explicitly, but consider the spread as translating into different initial phenotype distributions at primary and secondary sites. The different compartments in the model for each site represent epithelial, hybrid, and mesenchymal phenotypes. The solid arrows indicate competitive growth and phenotype transitions. Phenotype *i* grows at rate *r*_*i*_ and transitions to the adjacent more mesenchymal-like type at rate *T*_*EM*_ and to the adjacent more epithelial-like type at rate *T*_*ME*_. Resource competition is modeled with the term ${r}_{i}\frac{X}{K}$ where $X=\mathop{\sum }\nolimits_{j = 1}^{N}{x}_{j}$ is the total population abundance, and *K* is the carrying capacity. The epithelial phenotype can only transition to the more mesenchymal-like adjacent hybrid phenotype, and the mesenchymal phenotype can only transition to the epithelial-like adjacent hybrid phenotype; thus, the epithelial and mesenchymal phenotypes are the terminal phenotypes. Here, we show only one hybrid phenotype, but we also investigate the effect of a larger number of hybrid phenotypes.

Using a dynamical model of the abundances, *x*_*i*_, of *N* distinct phenotypes with linearly decreasing growth rates along the epithelial-mesenchymal trait axis, *i* = 1, 2, …, *N*, we track the temporal phenotype dynamics before, during and after treatment (see Fig. 1). We implement competition between phenotypes by assuming a shared carrying capacity *K*. We include transitions to an adjacent mesenchymal-like phenotype *T*_*EM*_ and adjacent epithelial-like phenotype *T*_*ME*_. We propose that a factor can control the balance between these transitions *T*_*EM*_ and *T*_*ME*_. The concentration of this transition-modulating factor can be mapped to the transition bias *λ*. The relation between growth and speed of phenotype transition is encoded by transition speed *c*. Further, we investigate growth-dependent treatment *m*_*D*_, i.e., chemotherapy, and growth-independent treatment *m*_*I*_, i.e., immunotherapy, to explore treatments and administration sequences. For a detailed description of the modeling assumptions please see the Section Model.

### Phenotype transitions generate and maintain heterogeneity

Assuming that cells can transition into adjacent epithelial or mesenchymal phenotypes generates a phenotype distribution of abundances along the epithelial-mesenchymal trait axis. Without treatment, *m*_*D*_ = *m*_*I*_ = 0, the model (see Section Model) has two equilibria. One equilibrium is the state where the tumor vanishes, *x*_*i*_ = 0 for *i* = 1, 2, …, *N*. This equilibrium is unstable, implying that a tumor can always progress and increase in abundance without treatment. The second equilibrium is the state described by

1 $${x}_{i}^{* }=K\frac{{(1-\lambda )}^{N-i}\,{(1+\lambda )}^{i-1}}{\mathop{\sum }\nolimits_{j = 1}^{N}{(1-\lambda )}^{N-j}\,{(1+\lambda )}^{j-1}}\qquad \,{{\mbox{for}}}\,\,i=1,2,\ldots ,N.$$

This equilibrium is stable and describes the coexistence of all phenotypes (see Supplementary Section Stability of the coexistence equilibrium for a proof of global stability). This stability implies that phenotypic heterogeneity is generated and maintained in tumor populations featuring phenotype transitions. Patient-derived breast cancer cell populations exhibit similar behavior in-vitro^34,35^.

The sum of all phenotype abundances at the coexistence equilibrium is *K*. The stable distribution of phenotypes at the coexistence equilibrium depends on the transition bias *λ* and the number of phenotypes *N*. Notably, the stable phenotype distribution does not depend on the growth rate as the transition speed is assumed to be phenotype-independent (see Section Model for details). Thus, only the transitions and not the growth dynamics decide the phenotype abundances at equilibrium. Additionally, this growth independence shows that the particular choice of the decline of growth rates from epithelial to mesenchymal phenotypes does not affect the equilibrium phenotype distribution.

### Transition bias controls the stable phenotype distribution

**Fig. 2:**
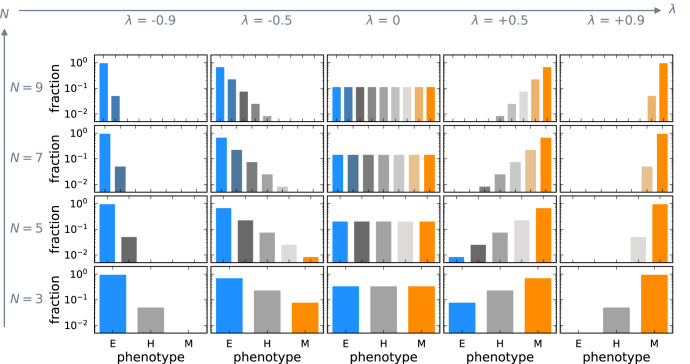
Phenotypic plasticity creates a stable phenotype distribution. Each panel shows the distribution of phenotypes (Eq. (1)) relative to the carrying capacity *K* for a fixed value of the transition bias *λ* and the number of phenotypes *N*. The stable phenotype distribution changes with the transition bias *λ* but remains qualitatively unaffected by changing the number of phenotypes *N*. The stable distribution is uniform when there is no transition bias to either epithelial or mesenchymal-like phenotypes, i.e., *λ* = 0. *λ* < 0 depicts a transition bias towards epithelial-like phenotypes and leads to a relative increase in epithelial cells. Conversely, *λ* > 0 results in a transition bias towards mesenchymal-like phenotypes and causes a relative increase in mesenchymal cells.

When cells change to a more epithelial or mesenchymal phenotype with the same probability, i.e., when there is no transition bias (*λ* = 0), all phenotypes are at equal abundance and the stable distribution is a uniform distribution (Fig. 2). The mesenchymal phenotype (M) is the most abundant when there is a transition bias towards the mesenchymal phenotypes (*λ* > 0). On the other hand, for a transition bias towards the epithelial phenotypes (*λ* < 0), the epithelial phenotype (E) is the most abundant. The phenotypic heterogeneity of the tumor population is highest when there is no transition bias and *λ* = 0 (Supplementary Fig. 1). A high variance corresponds to the presence of a lot of different phenotypes reducing the efficacy of standard cancer treatments. The heterogeneity decreases and vanishes at the bias extremes *λ* → ± 1 where transitions become unidirectional.

The presence of phenotypes far off the average population phenotype with very low abundances is captured by the third central moment of the phenotype distribution. These phenotypes are essential for residual disease in some cancers, and thus an important potential treatment target. We find that the third central moment is an odd function of the transition bias, vanishing at *λ* = 0 and at high transition bias (Supplementary Fig. 1). The number of phenotypes *N* quantitatively affects the moments of the stable phenotype distribution but does not affect their shapes qualitatively (Supplementary Fig. 1). We will thus fix *N* = 3 phenotypes in the remainder of the study for illustration.

### Transition speed and rate of approach to equilibrium

**Fig. 3:**
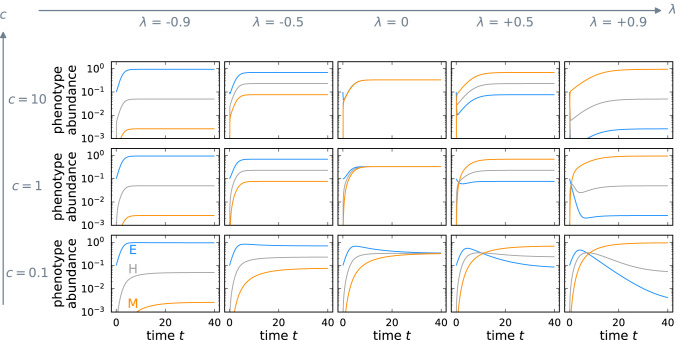
Transition speed determines the rate of approach to the stable phenotype distribution. The panels show the approach to the stable phenotype distribution from the same initial condition, $({x}_{1},{x}_{2},{x}_{3})=(\frac{1}{10},0,0)$, for different combinations of transition speed *c* and transition bias *λ*. The transition speed *c* sets the pace of the transition dynamics relative to the growth dynamics. For any transition bias *λ*, the time to reach the stable phenotype distribution decreases as the transition speed increases.

While the transition bias *λ* determines the stable phenotype distribution, the transition speed *c* affects how fast the stable phenotype distribution is approached. The model consists of two processes; logistic growth and transitions, where the parameter *c* scales the transition propensity relative to the growth rate. When transitions are faster than growth, *c* > 1, the population first reaches the stable phenotype distribution and then approaches the carrying capacity. When transitions are slower than growth, *c* < 1, the population first reaches the carrying capacity and then equilibrates to the stable phenotype distribution (Fig. 3).

### Initial phenotype distribution determines phenotype shifts

**Fig. 4:**
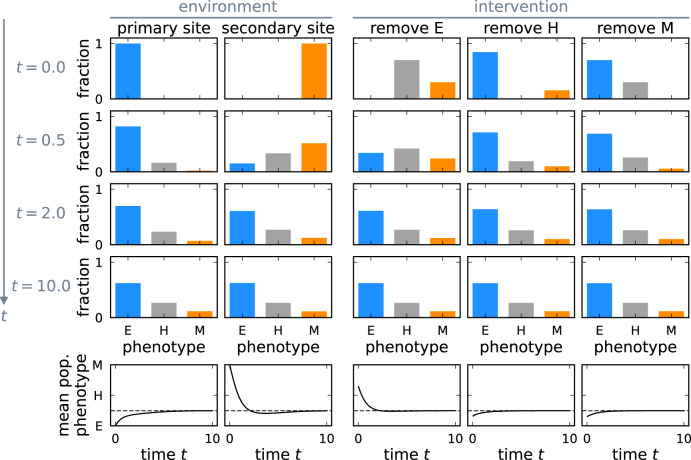
Plasticity-driven approach to stable phenotype distribution from different initial conditions. Depending on the initial condition, the approach to the stable phenotype distribution proceeds along different paths (top four rows, the vertical axis represents time). The mean population phenotype over time is shown in the last row with its asymptote (dashed line). The first column shows the phenotype dynamics of a plastic tumor at the primary site, where it originates only from epithelial cells. The second column represents the growth of a metastasis after mainly mesenchymal cells have arrived at the secondary site. The last three columns show the dynamics after a hypothetical intervention that removes all epithelial, all hybrid, or all mesenchymal cells. In all cases, the tumor approaches the stable phenotype distribution; however, different initial conditions lead to shifting the average phenotype in different directions.

The stability of the coexistence equilibrium induces phenotype shifts similar to epithelial-mesenchymal and mesenchymal-epithelial transitions. The primary site of a tumor at an early stage is composed mainly of proliferative epithelial cells. Thus, at the primary site, the stable phenotype distribution is approached by a relative increase in the abundance of mesenchymal-like cells (Fig. 4, first column). The secondary tumor site is initially founded by mesenchymal-like cells due to the selection pressure for invasive properties during dissemination. Therefore, conversely to the primary site, the stable equilibrium is approached by a relative increase of epithelial-like cells at the secondary site (Fig. 4, second column) during metastasis formation.

After a perturbation from the coexistence equilibrium, e.g., by treatment, the stable phenotype distribution is restored, compensating the effect of the perturbation (Fig. 4, columns three to five). Hence, the phenotypically plastic tumor cell population can maintain heterogeneity. Our model thus exhibits site- and treatment-specific phenotype shifts that result only from the selection during dissemination or treatment. These shifts do not require specific microenvironmental factors, although the microenvironment is a crucial component contributing to the initial conditions that determine the direction of the shift.

### Treatment affects the phenotype distribution of the tumor

**Fig. 5:**
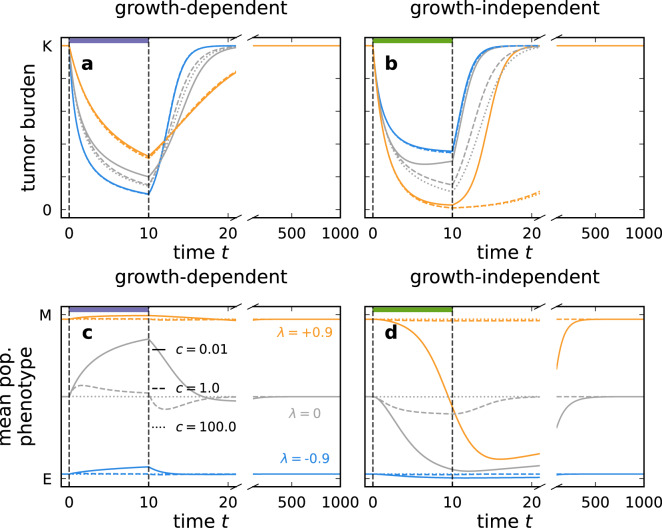
Growth-dependent and growth-independent treatment can transiently alter the phenotype distribution of a tumor. During treatment, the tumor burden shrinks (**a**, **b**), and the mean phenotype of the tumor changes if the transition speed *c* is small but restores to the untreated mean phenotype value after treatment (**c**, **d**). Treatment is applied between *t* = 0 and *t* = 10. Afterward, regrowth is tracked until *t* = 1000. The violet and green horizontal bars indicate growth-dependent and growth-independent treatments. Growth-dependent treatment shifts the mean of the phenotype distribution towards the mesenchymal phenotype as it exerts higher mortality on epithelial cells. Conversely, growth-independent treatment shifts the mean towards the epithelial phenotype as epithelial cells compensate for the mortality by faster growth.

Next, we tested how the phenotype distribution changes during and after growth-dependent and growth-independent treatment (Fig. 5). Growth-dependent treatment targets proliferating cells, thus epithelial cells experience higher treatment-induced mortality (Fig. 5a), and the mean of the distribution shifts towards the mesenchymal phenotype (Fig. 5c). Growth-independent treatment targets all phenotypes equally, not changing the phenotype distribution directly. The room for growth is filled by epithelial-like cells as they grow fast during treatment as the tumor is not at carrying capacity (Fig. 5b). Thus, during growth-independent treatment, the phenotype average shifts towards the epithelial phenotype, especially, if transitions are slow compared to growth dynamics (*c* < 1, Fig. 5d). After treatment, the phenotype composition returns to the stable distribution that is purely determined by the transition bias (Eq. (1)). The restoration of the stable phenotype distribution is driven by regrowth of the suppressed phenotypes and therefore occurs considerably slower after growth-independent treatment, i.e., in the direction of a frequency increase of mesenchymal cells (Fig. 5d). Such relapses at different rates after distinct treatment types align with our previous findings^24^ and clinical observations (Section 7 in Chitadze et al.^40^).

### Tumor with high transition speed is vulnerable to treatment

**Fig. 6:**
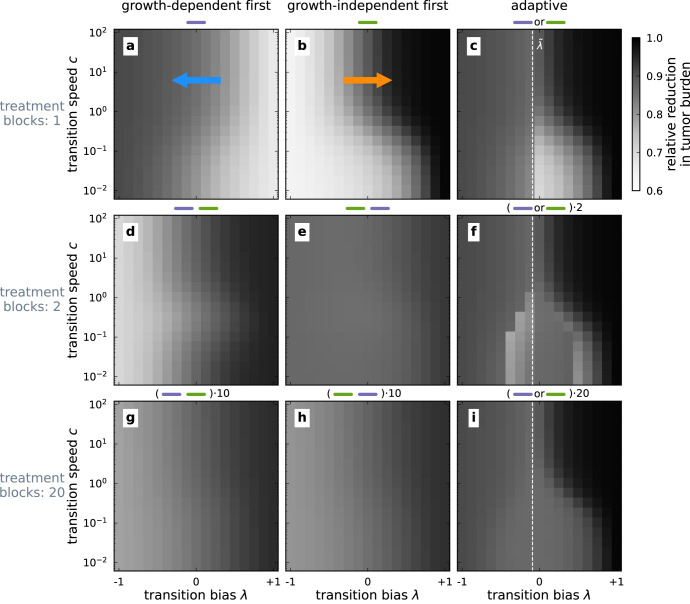
Reduction in tumor burden for different sequential treatment schemes. The reduction in tumor burden relative to the carrying capacity $\frac{K-X}{K}$ is indicated by the brightness gradient for different combinations of transition bias *λ* and transition speed *c*. Here, *X* is the sum of phenotype abundances at the end of treatment duration (*N* = 3). Darker colors represent a higher reduction and, thus, a better outcome. We evaluate the effect of splitting the treatment period into multiple treatment blocks (rows) and investigate different treatment schemes with either predefined or adaptive treatment sequences (columns). The white dashed line indicates a decision boundary $\tilde{\lambda }$ for the adaptive treatment, which is obtained by comparing the mortality of the two treatment types (see text). Adjuvant therapy can alter the phenotype transitions, which in our model translates to changes, for example, to the transition bias *λ*, indicated by the arrows in panels **a** and **b**.

Exploring a broader range of values for transition bias and speed, we find that the effect of growth-dependent and growth-independent treatment indeed depends strongly on transition bias and speed (Fig. 6). When a treatment type exerts a particularly high mortality on the most abundant tumor phenotype we refer to the treatment as a phenotype-matched treatment. For instance, growth-dependent treatment exerts the highest mortality on epithelial tumors, *λ* → − 1, whereas growth-independent treatment is more phenotype-matched to mesenchymal tumors, *λ* → 1. We find that one-block treatment schemes are most effective, i.e. achieve the strongest reduction in tumor burden, when they are phenotype-matched (Fig. 6a, b). Applying them for the whole treatment duration thus results in the highest reduction of tumor burden. Higher transition speeds *c* cause a faster replenishment of treatment-sensitive phenotypes from less sensitive phenotypes, entailing a cost of phenotypic plasticity. Thus, treatment efficacy improves for higher transition speeds for single-block treatments.

When the phenotype distribution is known, the adaptive treatment scheme can be applied (see Section Treatment types and schemes for a detailed description). This treatment scheme selects the treatment type that exerts the higher mortality on the tumor, given the phenotype distribution at the time of treatment choice. We assume that initially, the phenotype distribution has reached its equilibrium given by Eq. (1), which is determined mainly by the transition bias *λ*. For the one-block adaptive treatment scheme only at the beginning of the treatment phase a treatment type has to be determined. Thus, the treatment type decision in the adaptive one-block treatment scheme depends only on the transition bias *λ*. The decision boundary between applying the growth-dependent and the growth-independent treatment in terms of transition bias can be found by comparing the mortality exerted by the two treatment types. The total mortality of growth-dependent treatment $\mathop{\sum }\nolimits_{i = 1}^{N}{m}_{D}\,{r}_{i}\,{x}_{i}^*$ and growth-independent treatment $\mathop{\sum }\nolimits_{i = 1}^{N}{m}_{I}\,{x}_{i}^*$ are equal when $\mathop{\sum }\nolimits_{i = 1}^{N}{m}_{D}\,{r}_{i}\,{x}_{i}^{* }-{m}_{I}\,{x}_{i}^{* }=0$. This is a polynomial in the transition bias *λ* with the relevant root $\tilde{\lambda }\in [-1,1]$ setting the decision boundary between the two treatment types. For our parameters and assumptions on the growth rates of the phenotypes (Table 1), the switch occurs at $\tilde{\lambda }\approx -0.089$. Consequently, in the one-block adaptive treatment (Fig. 6c), the reductions of more epithelial tumors with $\lambda \,<\, \tilde{\lambda }$ are identical to reductions obtained for the growth-dependent treatment type. More mesenchymal tumors with $\lambda \,>\, \tilde{\lambda }$ are affected by one-block adaptive treatment identically to the growth-independent treatment.

**Table 1:** Symbols, variables and reference parameters.

Symbol	Description	Value
*x*_*i*_	Abundance of *i*^*th*^ tumor phenotype	
*X*	Sum of all phenotype abundances	$\mathop{\sum }\limits_{i = 1}^{N}{x}_{i}$
*N*	Number of tumor phenotypes	3
*r*_*i*_	Growth rate of *i*^*th*^ phenotype	${r}_{1}-({r}_{1}-{r}_{N})\,\frac{i-1}{N-1}$
*r*_1_	Growth rate of epithelial cells	1
*r*_*N*_	Growth rate of mesenchymal cells	${\displaystyle{\frac{1}{5}}}$
*K*	Carrying capacity of a site	1
*T*_*EM*_	Transition rate of *i* → *i* + 1	*c* (1 + *λ*) *r*_1_
*T*_*ME*_	Transition rate of *i* → *i* − 1	*c* (1 − *λ*) *r*_1_
*c*	Transition speed	1
*λ*	Transition bias	0
*m*_*D*_	Growth-dependent treatment intensity	1
*m*_*I*_	Growth-independent treatment intensity	≈ 0.64

Deviations from these values are reported where applicable.

### The effect of sequential multi-block treatment schemes

To explore the potential of leveraging the treatment-induced phenotype changes, we also investigated sequential treatment patterns that consist of multiple treatment blocks. In the multi-block schemes (see Supplementary Fig. 2 for tumor burden and mean phenotype dynamics of the two-block scheme), the application duration of the optimal treatment is reduced, thus resulting in a less effective treatment of tumors with a high transition bias (Fig. 6d, e). Frequent treatment type alterations result in an intermediate tumor burden reduction across trait space. Although frequent treatment type alteration is not the most effective strategy, it may be desirable when the characteristics of a tumor, such as transition bias *λ* and transition speed *c*, are unknown (Fig. 6g, h).

For the two-block adaptive treatment, the tumor reduction pattern is more intricate (Fig. 6f). At $\lambda \,>\, \tilde{\lambda }$, the mortality exerted by growth-independent treatment is higher; thus, growth-independent treatment is chosen as the first treatment type. At large transition speed *c* and high transition bias *λ*, growth-independent treatment is also chosen for the second treatment block. Only for a transition bias slightly exceeding $\tilde{\lambda }$ and small *c*, a treatment switch occurs, and the tumor burden reduction is higher than in the single-block scheme. For $\lambda \,<\, \tilde{\lambda }$, the first treatment type is growth-dependent treatment. Only for small *c* and close to $\tilde{\lambda }$ the treatment type is switched after half the treatment period, which interestingly worsens the treatment outcome compared to a single block of only growth-dependent treatment. This choice is possible as the decision criterion for treatment choice is the instantaneous mortality and does not account for the potential future tumor size or the future phenotype distribution, or mortality integrated over time. Since the growth-independent treatment exerts no selection pressure against the proliferative phenotype, switching to this treatment type comes at the risk that soon after the switch, the phenotype distribution will have shifted to a larger epithelial proportion, where the growth-independent treatment is less effective than the growth-dependent treatment. Increasing the number of treatment blocks mediates this risk and achieves the highest tumor burden reduction for small *c* and intermediate *λ* in the adaptive treatment scheme. Different modeling assumptions (unequal competitiveness of phenotypes or phenotype-dependent transition speed) change the effect of treatment only quantitatively (see Supplementary Sections Phenotype-dependent transition speed, and Unequal competition, Supplementary Figs. 5, 8).

### The potential of adjuvant treatment

Modifying the process of phenotype transition with adjuvant therapies promises to alter the phenotype distribution of a tumor, thus, rendering the tumor more vulnerable to chemo- or immunotherapy. Phenotype transitions may be modulated, equivalent to changing transition bias *λ*, by an adjuvant drug that affects the gene regulatory network of epithelial-mesenchymal transitions^25,26,29^. Figure 6 indicates how modifying the transition bias *λ* with adjuvant drugs can affect the reduction in tumor burden. We find that the tumor burden is reduced more strongly when a single-block growth-dependent treatment is accompanied by an adjuvant drug that makes the tumor more epithelial by decreasing the transition bias *λ* (arrow in Fig. 6a). Contrastingly, the effect of the single-block growth-independent treatment can be enhanced by an adjuvant drug that makes the tumor more mesenchymal by increasing the transition bias *λ* (arrow in Fig. 6b). For multi-block treatment schemes, we find that increasing transition bias *λ* generally also increases the reduction in tumor burden, albeit this effect depends on the exact pair of transition bias *λ* and speed *c* for the adaptive treatment. Adjuvant therapies that increase the transition speed *c* increase the treatment effect for a single-block treatment, but for two treatment blocks the outcome is difficult to analyze due to the dependence on the treatment sequence, transition bias *λ* and speed *c*.

## Discussion

Cancer cell plasticity increases tumor heterogeneity, facilitates metastasis formation, and often hinders treatment approaches^41^. We formulated a mathematical model of phenotypic plasticity that motivates a stable heterogeneous phenotype distribution, mainly determined by the relative propensities of phenotype transitions. We found that the heterogeneity generated by this inherent plasticity can explain the transitions between epithelial and mesenchymal phenotypes, which are known to facilitate cancer progression and metastasis^4,6,13,14,38,42^. Our model provides an alternative hypothesis for the factors driving such transitions, not necessarily relying on the microenvironment, yet sensitive to microenvironmental changes. The stability of a heterogeneous phenotype distribution implies that perturbations to this distribution will be reverted. We discuss the consequences of this equilibrium for primary and secondary tumors and their treatment in this section.

### Phenotypic plasticity drives phenotype shifts at the primary and secondary tumor sites

During metastasis formation, one prominent phenotype transition in epithelial cancer cells is the epithelial-mesenchymal transition^14,23^. Tumors of epithelial origin gain heterogeneity by transitioning to a more mesenchymal phenotype. Selection for motility and invasiveness during the dissemination of cancer cells increases the frequency of the mesenchymal phenotype in the circulating cancer cells. Therefore, the arriving population at the secondary site is mainly constituted of mesenchymal phenotype and gains heterogeneity by recovering more epithelial phenotypes. Thus, we find that phenotypic plasticity can be responsible for both the epithelial-mesenchymal transition at the primary site and the mesenchymal-epithelial transition at the secondary site. However, microenvironmental differences undoubtedly exacerbate phenotypic changes during the dissemination from primary to secondary site^8^. Indeed, we can capture much more pronounced phenotype changes by assuming different transition biases *λ*_1_ ≠ *λ*_2_ at primary and secondary sites, resulting in differing stable phenotype distributions.

### Moments of the phenotype distribution guide the choice of phenotype-matched treatment

We investigated growth-dependent and growth-independent treatment types, capturing population-level mechanisms of chemo- and immunotherapy. We found that the transition bias, and thus the shape of the stable phenotype distribution, determines the most effective treatment type and sequence of different treatment types. Our model exhibits a higher efficiency of growth-dependent treatment for a transition bias towards epithelial phenotypes. Growth-independent treatment becomes more effective for a transition bias towards mesenchymal phenotypes. This connection between treatment effect and the shape of the phenotype distribution can be understood better by considering the moments of the phenotype distribution. Narrow phenotype distributions are well characterized by their mean, which can be used to match treatment type and phenotype. For example, chemotherapy will be the most effective when there are only proliferative cells. Such a phenotype-matched treatment will perform well for homogeneous tumors in the absence of mismatching terminal phenotypes. To implement this strategy in clinical practice, a thorough characterization of the tumor tissue would be necessary. With the identification of the dominating phenotypes a patient may be stratified for phenotype-matched treatment. In-vitro analysis of patient-derived tumor tissues may be used to test and determine the most effective treatment for the patient.

However, tumors are often heterogeneous, weakening their treatment response^43,44^. Interestingly, the third central moment of the phenotype distribution captures the mismatch between a treatment type and the abundance of phenotypes that are not matched by the treatment, as they are far off the mean of the phenotype distribution. When matching the treatment to the mean of the phenotype distribution, an increasing absolute third central moment thus signals the onset of relapse from mismatched phenotypes. In our model, the phenotypic heterogeneity of the stable phenotype distribution is maximal when there is no transition bias, i.e., *λ* = 0. The mismatch between targeted phenotype and unmatched phenotypes, captured by the third central moment, is largest at intermediate transition bias. Accordingly, our model suggests the existence of a sweet spot for controlling both heterogeneity and mismatched terminal phenotypes at extreme transition biases *λ* → ± 1, where variance and absolute third central moment are simultaneously minimized.

### Driving factors of the phenotype transition control the phenotype distribution

Recently, several studies suggested treatment strategies for phenotypically heterogeneous tumors^24,35,36,45^. These strategies aim to remove or reduce the tumor burden and delay tumor relapse. However, these studies do not target or capitalize on the source of the heterogeneity. The interaction between treatment and phenotype dynamics can potentially be exploited during cancer treatment as the transition bias and, therefore, the stable phenotype distribution can be modulated by an adjuvant therapy. In the context of epithelial-mesenchymal plasticity, the driving factors of the epithelial-mesenchymal transition and the mesenchymal-epithelial transitions are known^17,32^. Altering the concentrations of these driving factors in the tumor microenvironment or perturbing the relevant gene regulatory network would thus translate to changing transition bias *λ* and speed *c* which are decisive for the phenotype transition process. For instance, frequent phenotype switching is costly in rarely changing environments^46^. Similarly, in our simulations high transition speed *c* improves treatment efficacy. Experimental testing could determine whether indeed faster transition speed poses a cost and can be exploited in clinical settings. Modulation of transition bias *λ* has also been shown with TGF-*β* blockage^25,26,28^. Therefore, it may be possible to regulate tumor phenotypes, reduce metastasis, and achieve a better treatment response by controlling the transition bias with an adjuvant treatment. Of note, this reasoning applies also to tumor sites that have reached carrying capacity. Although the total abundances of such tumors may be static, the phenotype distribution can still change^47^. Also in our model, at carrying capacity, the population growth stops, but the transition dynamics can remain in action, capturing the potential turnover in tumor composition.

### Driving factors of phenotype transition may serve as a proxy for phenotype abundances

When measuring the concentrations of driving factors is more feasible than measuring the phenotype abundances, the concentrations could also be used to characterize tumor heterogeneity building on the decisive effect of such driving factors of the phenotype transitions. Hypothetically, in the case of TGF-*β* driven epithelial-mesenchymal transition, the tumor could be characterized by the concentration of TGF-*β* instead of quantifying the number of epithelial, hybrid, and mesenchymal cells. More generally, the state of the microenvironment or the relevant gene regulatory network can determine the phenotype distribution, and thus, be used to inform treatment decisions^29^.

Additionally, if available, the third central moment of the phenotype distribution, i.e., the abundance of mismatched phenotypes, can characterize the relapse. For example, consider a tumor of epithelial cells with a high third central moment. If this tumor is treated with growth-dependent treatment, then the relapse will initially be driven by the growth of mesenchymal cells, eventually, the epithelial cells will take over the population, leading to bi-phasic relapse dynamics^24,40^. The present model provides a link between the quantification of driving factors of phenotype transition, equivalent to the transition bias *λ*, and the third central moment of the phenotype distribution.

In this study, we formulated a mathematical model that quantitatively describes the transition between adjacent phenotypes within a population. Phenotype transitions appear in most forms of unicellular organisms. Our findings, suggesting targeting phenotype transitions to improve treatment are, hence, applicable beyond cancer, i.e., for pathogen-borne diseases such as bacterial infections. In the current study, we make assumptions that allow exposition of our findings, e.g. the phenotype transitions are not adaptive. Also, we do not model the spread between the primary and secondary cancer sites explicitly. Exchanging some of our simple assumptions for realism, we also discuss the case of unequal competition and growth-dependent transition speed in the Supplementary Section Model extensions. The case of unequal competition can be linked to frequency-dependent interactions between tumor phenotypes, which can be captured in the present model by unequal competition coefficients. There are compelling studies investigating frequency-dependent interactions^48–50^ with some quantifying them in patient-derived tumors^51,52^, which could affect treatment outcome and thus, the prognosis of the disease.

To summarize, the present model provides a microenvironment-independent explanation for observed phenotype changes during epithelial-mesenchymal and mesenchymal-epithelial transitions. We highlight the phenotype transitions in a heterogeneous population as a viable clinical target. By targeting the driving factors or the relevant gene regulatory network of the phenotype transition, we propose leveraging phenotypic plasticity. Forcing the tumor into a less heterogeneous state, this strategy may improve the treatment efficacy of chemo- and immunotherapies. Supporting the established idea of personalized treatments, our approach thus discusses a practical option to bring the concept into clinical practice.

## Methods

We investigate the onset of metastasis formation mediated by the plasticity between epithelial and mesenchymal phenotypes. We use an ecology-inspired approach to identify tumor phenotypes with species in an ecosystem, drawing on the usefulness of ecological concepts for understanding cancer biology^53,54^. We use the competitive Lotka–Volterra system^55,56^ with additional terms that capture transitions between phenotypes. We go beyond earlier approaches that featured two cell types^35,36^ and account for the recently established prominence of hybrid phenotypes characterized by both partial epithelial and mesenchymal markers^15^. This results in a multi-compartment model similar to those presented in Zhou et al.^33^ and Raatz et al.^24^, but with added competition and more flexibility in specifying the transition process. Using this model, we examine the epithelial-mesenchymal plasticity and characterize how different properties of the plastic transition shape the resulting tumor heterogeneity.

### Model

We represent the phenotypic heterogeneity as a collection of *N* different tumor phenotypes at different sites of cancer (Eq. (2)). These phenotypes range from the most epithelial phenotype, E, over *N* − 2 hybrid phenotypes H, to the most mesenchymal phenotype, M. We assume that all tumor phenotypes share resources at a given cancer site and that the tumor population follows logistic growth^57^. Additionally, we assume that the tumor cells are phenotypically plastic, i.e., they can change to an adjacent, more epithelial-like, or mesenchymal-like phenotype in bidirectional phenotype transitions.

We track the phenotype abundance *x*_*i*_ over time for each tumor phenotype *i*, *i* = 1, 2, …, *N*. Each cell of tumor phenotype *i* grows intrinsically at rate *r*_*i*_. The cells switch to the next functionally more mesenchymal phenotype, *i* → *i* + 1, at rate *T*_*EM*_, and to the next functionally more epithelial type, *i* → *i* − 1, at rate *T*_*ME*_. The positive transition terms represent additions to a phenotype from adjacent phenotypes. The negative transition terms account for the losses from transitions out of the current phenotype. Resource scarcity in the microenvironment induces a density-dependent, logistic competition captured by ${r}_{i}\,\frac{X}{K}$, where $X=\mathop{\sum }\nolimits_{i = 1}^{N}{x}_{i}$ is the total population abundance and *K* is the carrying capacity. We assume that all phenotypes are equally competitive for resources, i.e., the competition term is equal for all phenotypes. Relaxing this assumption does not affect our results qualitatively and is explored in the Supplementary Section Unequal competition.

Further, we assume linearly decreasing growth rates from epithelial to mesenchymal phenotypes, reflecting the higher proliferation rates of epithelial cells. We scale all growth rates relative to the epithelial growth rate *r*_1_, and express the growth rate for the *i*^*th*^ phenotype as ${r}_{i}={r}_{1}-({r}_{1}-{r}_{N})\,\frac{i-1}{N-1}$. Note that all the growth rates are equally distributed in the interval [*r*_1_, *r*_*N*_]. The independence of the equilibrium phenotype distribution (Eq. (1)) from the growth rates indicates that the particular choice of the growth rate decline does not affect our results qualitatively.

We assume that the transition rates are equal for all phenotypes and scaled to the epithelial growth rate *r*_1_ with a constant *c*, 0 ≤*c* < *∞*. Thus, transitions faster than proliferation, *c* > 1, can be interpreted as within-generation phenotypic plasticity, e.g. altered patterns of post-transcriptional regulation, and transitions slower than proliferation *c* < 1 can be attributed to transgenerational plasticity, such as epigenetic changes. We explore the effect of growth rate-dependent transition rates in the Supplementary Section Phenotype-dependent transition speed.

To account for a potential bias in the direction of plastic transitions, we introduce the transition bias *λ*, − 1 ≤ *λ* ≤ 1, which determines the probability of a cell switching to the adjacent epithelial or mesenchymal phenotype. A positive transition bias *λ* > 0 implies a bias towards becoming more mesenchymal, *λ* < 0 results in a higher probability of switching to a more epithelial phenotype. With these assumptions, the transition rates become *T*_*EM*_ = *c* (1 + *λ*) *r*_1_ and *T*_*ME*_ = *c* (1 − *λ*) *r*_1_. The parameters of our model are given in Table 1.

### Treatment types and schemes

To investigate the effect of drug treatment on phenotypically plastic tumor cell populations, we apply two different treatment types. The mortality exerted on the cells of a particular phenotype by the two treatment types either does or does not depend on the phenotype’s growth rate. The death rate exerted by growth-dependent treatment is *m*_*D*_ *r*_*i*_, and the death rate by growth-independent treatment is *m*_*I*_. To better compare the phenotype-distribution-dependent treatment effects, we chose *m*_*I*_ such that the reduction of tumor cell abundance at the end of the treatment duration is identical for both treatment types. To find *m*_*I*_, we use *c* = 1, *λ* = 0, *m*_*D*_ = 1, and numerically minimize the difference between the total abundances *X* at the end of treatment duration. Throughout this study, treatment is always applied for a fixed duration of 10 time units.

These assumptions result in a system of ordinary differential equations describing the change in tumor phenotype abundance,

2 $$\begin{array}{rcl} \frac{dx_1}{dt} & =& r_1\,x_1 \left(1 - \frac{X}{K} \right) + c\,r_1\left[(1-\lambda)\,x_2 - (1+\lambda)\,x_1\right] - (m_D\,r_1 + m_I)\,x_1 \\ \vdots && \\ \frac{dx_i}{dt} &=& \mathop{\underbrace{r_i\,x_i \left(1 - \frac{X}{K} \right)}}\limits_{\substack{{\rm{logistic}}\,{\rm{growth}}}} + \mathop{\underbrace{c\,r_1\left[(1+\lambda)\,x_{i-1} + (1-\lambda)\,x_{i+1} - 2\,x_i\right]}}\limits_{\substack{{\rm{transitions}}}} - \mathop{\underbrace{(m_D\,r_i + m_I)\,x_i}}\limits_{\substack{{\rm{treatments}}}}. \\ \vdots && \\ \frac{dx_N}{dt} &=& r_N\,x_N \left(1 - \frac{X}{K} \right) + c\,r_1\left[(1+\lambda)\,x_{N-1} - (1-\lambda)\,x_N\right] - (m_D\,r_N + m_I)\,x_N \end{array}$$

Different drug administration strategies are possible and of clinical interest. However, in current practice, mostly monotherapies or combined therapies are used, often leading to resistance due to static selection pressures. This motivates us to investigate sequential treatment schemes. The fixed treatment scheme either treats with the same drug throughout the treatment duration or alternates between blocks of different treatment types. The single-block treatment scheme applies either growth-dependent or growth-independent treatment. In the multi-block treatment schemes, we alternate between treatment types and investigate both cases of first treating with growth-dependent or first treating with growth-independent treatment. For the adaptive treatment scheme, at the start of each treatment block, the treatment type is chosen that achieves the higher instantaneous reduction of the whole tumor cell population for the given phenotype distribution^24^. The cancer cell mortality for growth-dependent treatment is $\mathop{\sum }\nolimits_{i = 1}^{N}{m}_{D}\,{r}_{i}\,{x}_{i}$ and the mortality exerted by growth-independent treatment is $\mathop{\sum }\nolimits_{i = 1}^{N}{m}_{I}\,{x}_{i}$. The comparison of these two terms determines the decision for the treatment type.

### Analysis and implementation

We performed linear and Lyapunov stability analysis to find the equilibria of Eqs. (2) and establish their stability^58^. The results of this analysis are presented in the Supplementary Section Stability of the coexistence equilibrium. For *N* = 2 and *N* = 3 stability was confirmed computationally (Supplementary Fig. 3), for *N* > 3 we show stability using Lyapunov stability analysis. Temporal dynamics of the model (Eqs. (2)) were obtained by numerical integration using the solve_ivp function from the Scipy library^59^ in Python (version 3.9^60^). Numpy^61^ and Matplotlib^62^ were used for computation and plotting.

### Reporting summary

Further information on research design is available in the Nature Research Reporting Summary linked to this article.

### Supplementary information

Supplementary Notes

Reporting Summary

## Data Availability

The figures and dataset generated in this study have been deposited in a zenodo repository with 10.5281/zenodo.7989753.

## Code availability

The relevant code to generate the figures and dataset can be found in a zenodo repository with 10.5281/zenodo.7989748.
