# Supplementary material for: Understanding and leveraging phenotypic plasticity during metastasis formation
Source: NPJ Syst Biol Appl. 2023 Oct 6;9:48. doi: 10.1038/s41540-023-00309-1 (PMC10558468; doi:10.1038/s41540-023-00309-1)
Supplement: Supplementary file 1 — Supplementary Notes [file 41540_2023_309_MOESM1_ESM.pdf]

Supplementary Notes

Supplementary Figures

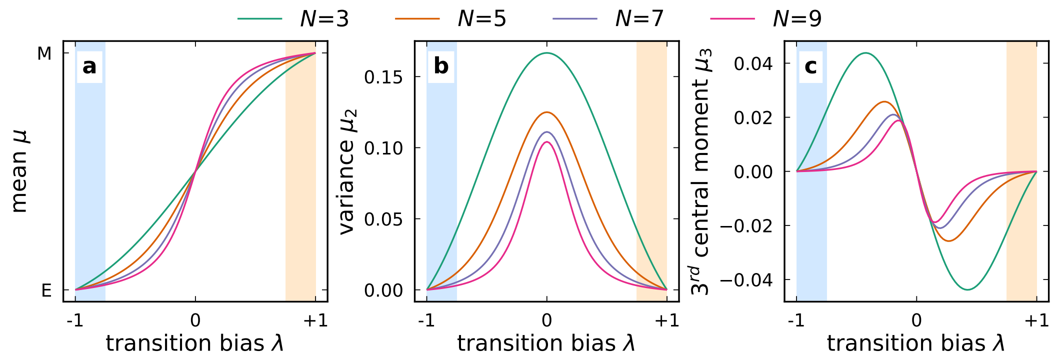

Supplementary Figure 1: The transition bias affects the phenotypic heterogeneity and abundances of mismatched phenotypes. The stable phenotype distribution $\Pr(i \mid \lambda, N) = \frac{x_i^*}{K}$, written as a function of transition bias λ for different numbers of phenotypes N, can be characterized by its moments. Wherever necessary, we scale the phenotype $i = 0, 1, \ldots, N-1$ to a trait axis, $\frac{i}{N-1} \in [0, 1]$, such that 0 always corresponds to epithelial (E) and 1 to mesenchymal (M). The first moment of the phenotype distribution is the mean $\mu = \sum_{i=0}^{N-1} \frac{i}{N-1} \frac{x_i}{X}$ (panel **a**). The nth central moment is $\mu_n = \sum_{i=0}^{N-1} \left(\frac{i}{N-1} - \mu\right)^n \frac{x_i}{X}$. Here, we show the 2nd (variance, panel **b**) and 3rd central moments (panel **c**) of the stable phenotype distribution. For measurements, label the phenotype as $i = 1, 2, \ldots, N$, measure their abundances x_i. The mean is the phenotype i weighted by the measured fraction or relative abundance $\frac{x_i}{X}$. **a** The mean allows the identification of phenotype-matched treatment, e.g., to select growth-dependent treatment for mainly epithelial tumors. **b** The variance represents phenotypic heterogeneity, which often impedes treatment success. **c** The third central moment is a proxy for the abundance of phenotypes far from the mean phenotype that are potentially unmatched by the phenotype-matched treatment. This measure can thus signal when the mean is an inaccurate representation of the phenotype distribution, for example, when it is strongly skewed. Thus, to maximize the efficacy of the phenotype-matched treatment it is favorable to minimize the heterogeneity and inaccuracy of the mean. High transition bias, $\lambda \to \pm 1$, minimizes both the heterogeneity and the inaccuracy of the mean, indicated by a combination of small variance and a small absolute third central moment. The high transition bias intervals are highlighted with colored background, where the color depicts the type of the most abundant phenotype.

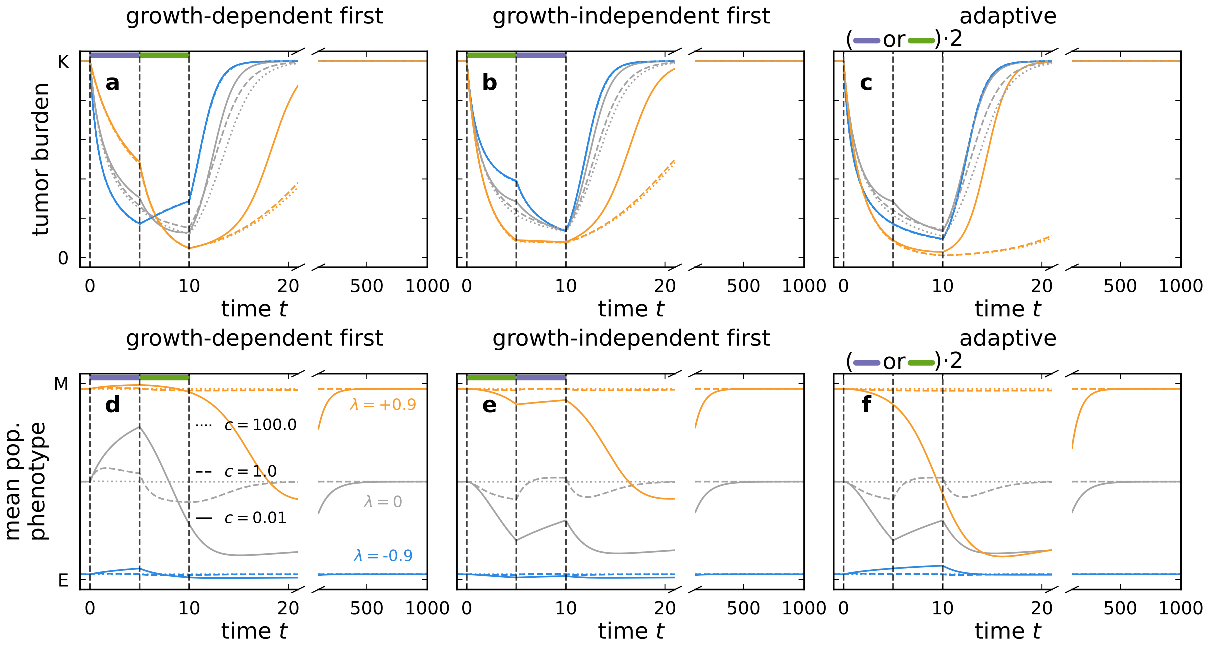

Supplementary Figure 2: Changing treatment types can increase the efficacy of treatment. The figure shows the effect of sequential two-block treatment on the total tumor abundance (first row) and mean phenotype (second row) during and after treatment for tumors with specific transition bias λ (line color) and transition speed c (line type). The growth-dependent treatment block is depicted by a violet bar on the top, and the growth-independent block is shown by a green bar. The first block is applied between $t = 0$ and $t = 5$, and the second block is applied between $t = 5$ and $t = 10$. After the treatment duration, the tumor regrowth is tracked until $t = 1000$. For the adaptive treatment scheme, the treatment type is chosen at the beginning of the block based on which type exerts the higher mortality on the present phenotype distribution. In practice, to find the best treatment type, we compare the mortalities exerted by growth-dependent $\sum_{i=1}^{N} m_D\ r_i\ x_i$, and growth-independent treatment $\sum_{i=1}^{N} m_I\ x_i$. Their difference decides the best treatment type, i.e., $\sum_{i=1}^{N} m_D\ r_i\ x_i - m_I\ x_i > 0$ implies that the growth-dependent treatment has higher mortality on the present phenotype distribution. Growth-dependent treatment is chosen for both blocks if the transition bias is low ($\lambda = -0.9$, blue lines). For unbiased, slow transitions ($\lambda = 0$ and $c = 0.01$), the treatment type switches from growth-independent to growth-dependent between the first and the second block (solid gray line). The growth-independent treatment type is applied in both treatment blocks for all other parameter combinations. Compared to single-block treatments (Fig. 5), tumor burden reductions at the end of the treatment phase can be higher or lower in two-block treatment schemes, depending on the transition bias and transition speeds, mirroring the complex patterns in Fig. 6. Two-block treatments are thus not always beneficial but can also reduce treatment efficacy if the treatment is not matched with the phenotype distribution.

Stability of the coexistence equilibrium

We investigate the stability of the coexistence phenotype distribution (Eq. (1)) analytically by linear stability analysis for $N = 2$ and $N = 3$[1]. For $N > 3$, we compute the eigenvalues using a Mathematica script (see Supplementary Material `linear_stability.nb`) and observe that they are negative for our parameter ranges. For an arbitrary number of phenotypes N, we use the Lyapunov stability theory [Chapter 5][2].

Two phenotypes $N = 2$

The following differential equations describe the system with two phenotypes without treatment.

$$\begin{aligned}
\frac{dx_1}{dt} &= r_1\, x_1 \left(1 - \frac{X}{K}\right) + c\, r_1\, [- \quad (1+\lambda)\, x_1 \quad +(1-\lambda)\, x_2] \\
\frac{dx_2}{dt} &= r_2\, x_2 \left(1 - \frac{X}{K}\right) + c\, r_1\, [\quad (1+\lambda)\, x_1 \quad -(1-\lambda)\, x_2],
\end{aligned} \tag{1}$$

where $X = x_1 + x_2$. The Jacobian $\mathbf{J}$ of the system at a point (x_1, x_2) is

$$\mathbf{J} = \begin{bmatrix} r_1\ \left(1 - \frac{X}{K}\right) - r_1 \frac{x_1}{K} - c\, r_1\ (1+\lambda) & -r_1 \frac{x_1}{K} + c\, r_1\ (1-\lambda) \\ -r_2 \frac{x_2}{K} + c\, r_1\ (1+\lambda) & r_2\ \left(1 - \frac{X}{K}\right) - r_2 \frac{x_2}{K} - c\, r_1\ (1-\lambda) \end{bmatrix}.$$

Evaluating the Jacobian at the coexistence equilibrium, $X = K$, $x_1^* = \frac{1}{2}K(1-\lambda)$, and $x_2^* = \frac{1}{2}K(1+\lambda)$ we get

$$\mathbf{J}^* = \begin{bmatrix} -r_1 \frac{(1-\lambda)}{2} - c\, r_1\ (1+\lambda) & -r_1 \frac{(1-\lambda)}{2} + c\, r_1\ (1-\lambda) \\ -r_2 \frac{(1+\lambda)}{2} + c\, r_1\ (1+\lambda) & -r_2 \frac{(1+\lambda)}{2} - c\, r_1\ (1-\lambda) \end{bmatrix}. \tag{2}$$

Given that the growth rates are positive by definition $r_i > 0$ and transition bias $\lambda \in [-1, 1]$, both the diagonal elements of this matrix are negative, and so must be its trace τ. For exposition, we use specific parameter values $r_1 = 1$, and $r_2 = \frac{1}{5}$, then the determinant Δ of the Jacobian at the coexistence fixed point $\mathbf{J}^*$ is

$$\Delta = \frac{4c(3 - 2\lambda)}{10}. \tag{3}$$

Since transition speed c is always positive by definition the determinant Δ of the Jacobian $\mathbf{J}^*$ is always positive. The stability of this 2D system can be determined from the trace-determinant plane[1]. The trace is always negative $\tau < 0$, and the determinant is always positive $\Delta > 0$. Therefore, the system is linearly stable at the coexistence fixed point.

Three phenotypes $N = 3$

The following differential equations describe the system with three phenotypes where $X = x_1 + x_2 + x_3$.

$$\begin{aligned}
\frac{dx_1}{dt} &= r_1\, x_1 \left(1 - \frac{X}{K}\right) + c\, r_1\, [-\ (1+\lambda)\, x_1 \qquad\qquad +(1-\lambda)\, x_2] \\
\frac{dx_2}{dt} &= r_2\, x_2 \left(1 - \frac{X}{K}\right) + c\, r_1\, [\ \ (1+\lambda)\, x_1 - 2\, x_2 \quad +(1-\lambda)\, x_3] \\
\frac{dx_3}{dt} &= r_3\, x_3 \left(1 - \frac{X}{K}\right) + c\, r_1\, [\ \ (1+\lambda)\, x_2 \qquad\qquad -(1-\lambda)\, x_3]
\end{aligned} \tag{4}$$

The Jacobian $\mathbf{J}$ of the system at a point (x_1, x_2, x_3) is

$$\mathbf{J} = \begin{bmatrix} r_1\,(1-\frac{X}{K}) - r_1\,\frac{x_1}{K} - c\,r_1\,(1+\lambda) & -r_1\,\frac{x_1}{K} + c\,r_1\,(1-\lambda) & -r_1\,\frac{x_1}{K} \\ -r_2\frac{x_2}{K} + c\,r_1\,(1+\lambda) & r_2(1-\frac{X}{K}) - r_2\frac{x_2}{K} - 2\,c\,r_1 & -r_2\frac{x_2}{K} + c\,r_1\,(1-\lambda) \\ -r_3\frac{x_3}{K} & -r_3\frac{x_3}{K} + c\,r_1\,(1+\lambda) & r_3(1-\frac{X}{K}) - r_3\frac{x_3}{K} - c\,r_1\,(1-\lambda) \end{bmatrix}.$$

At the coexistence equilibrium, $X = K$, $x_1^* = \frac{1}{\lambda^2+3}K(1-\lambda)^2$, $x_2^* = \frac{1}{\lambda^2+3}K(1-\lambda)(1+\lambda)$, and $x_3^* = \frac{1}{\lambda^2+3}K(1+\lambda)^2$, substituting this to get the Jacobian $\mathbf{J}^*$ at the coexistence fixed point simplifies to

$$\mathbf{J}^* = \begin{bmatrix} -r_1\,\frac{(1-\lambda)^2}{(\lambda^2+3)} - c\,r_1\,(1+\lambda) & -r_1\,\frac{(1-\lambda)^2}{(\lambda^2+3)} + c\,r_1\,(1-\lambda) & -r_1\,\frac{(1-\lambda)^2}{(\lambda^2+3)} \\ -r_2\frac{(1-\lambda)(1+\lambda)}{(\lambda^2+3)} + c\,r_1\,(1+\lambda) & -r_2\frac{(1-\lambda)(1+\lambda)}{(\lambda^2+3)} - 2\,c\,r_1 & -r_2\frac{(1-\lambda)(1+\lambda)}{(\lambda^2+3)} + c\,r_1\,(1-\lambda) \\ -r_3\frac{(1+\lambda)^2}{(\lambda^2+3)} & -r_3\frac{(1+\lambda)^2}{(\lambda^2+3)} + c\,r_1\,(1+\lambda) & -r_3\frac{(1+\lambda)^2}{(\lambda^2+3)} - c\,r_1\,(1-\lambda) \end{bmatrix}. \tag{5}$$

For simplicity, we continue with $r_1 = 1$, $r_2 = \frac{3}{5}$, $r_3 = \frac{1}{5}$, and $c = 1$ to get the characteristic polynomial $P(x)$. This characteristic polynomial of the Jacobian $\mathbf{J}^*$ is a 3rd order polynomial of the form $P(x) = x^3 + a_2x^2 + a_1x + a_0$ where

$$\begin{aligned}
a_2 &= \frac{5\lambda^4 + 42\lambda^2 - 32\lambda + 81}{(\lambda^2+3)\,(3\lambda^2 - 8\lambda + 9)} = \frac{P_4(\lambda)}{P_1(\lambda)\ P_2(\lambda)} \\
a_1 &= \frac{23\lambda^2 - 8\lambda + 69}{(\lambda^2+3)\,(3\lambda^2 - 8\lambda + 9)} = \frac{P_3(\lambda)}{P_1(\lambda)\ P_2(\lambda)} \\
a_0 &= \frac{5}{3\lambda^2 - 8\lambda + 9} = \frac{5}{P_2(\lambda)}
\end{aligned}$$

Now, according to the Routh-Hurwitz stability criterion[3], the system is stable if and only if a_2, a_1, and a_0 are positive and $a_2\ a_1 - a_0 > 0$. Above, we have defined four polynomials in λ. To show that a_2, a_1, and a_0 are positive, we determine the range of each polynomial for $\lambda \in [-1, 1]$. $P_1(\lambda) = \lambda^2 + 3$ is always positive for any real transition bias. The derivative of $P_2(\lambda) = 3\lambda^2 - 8\lambda + 9$ is $P_2'(\lambda) = 6\lambda - 8$ and P_2' vanishes at $\lambda = \frac{4}{3}$. As $P_2(\lambda)$ is a parabola opening upwards, $P_2(\frac{4}{3}) = \frac{11}{3}$ is the minimum, and $P_2(\lambda)$ is positive for any real transition bias. The derivative of $P_3(\lambda) = 23\lambda^2 - 8\lambda + 69$ is $P_3'(\lambda) = 46\lambda - 8$ and $P_3'(\lambda)$ vanishes at $\lambda = \frac{4}{23}$. As also $P_3(\lambda)$ is a parabola opening upwards, $P_3(\frac{4}{23}) = \frac{1571}{23}$ is a minimum, and also $P_3(\lambda)$ is positive for a real transition bias.

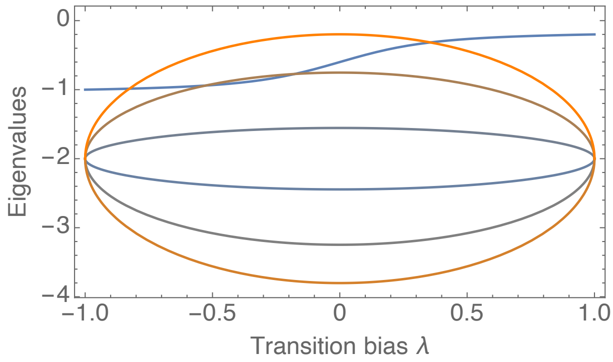

Supplementary Figure 3: The coexistence phenotype distribution is locally stable for any number of phenotypes. The Jacobian matrix of the system Eq. (2) is evaluated at the coexistence phenotype distribution, $x_i^* = K\frac{(1-\lambda)^{N-i}\ (1+\lambda)^{i-1}}{\sum_{j=1}^{N}(1-\lambda)^{N-j}\ (1+\lambda)^{j-1}}$, with $r_1 = 1$, $c = 1$. Each curve represents one eigenvalue of the Jacobian matrix. For the number of phenotypes $N = 7$, all 7 eigenvalues of the Jacobian matrix at the coexistence phenotype distribution are negative. Thus, the coexistence phenotype distribution is locally stable.

The first derivative of $P_4(\lambda) = 5\lambda^4 + 42\lambda^2 - 32\lambda + 81$ is $P_4'(\lambda) = 20\lambda^3 + 84\lambda - 32$ and has a unique real root $\lambda^* \approx 0.37$. The second derivative of $P_4(\lambda)$ is $P_4''(\lambda) = 60\lambda^2 + 84$ which is always positive for a real transition bias λ. Since $P_4''(\lambda)$ has no roots there is no inflection and $P_4(\lambda)$ is always concave up. Thus, $P_4(\lambda^*) \approx 75$ is the minimum as the curvature is always positive, and $P_4(\lambda)$ is positive for a real transition bias.

Therefore, a_2, a_1, and a_0 are positive for a transition bias $-1 < \lambda < 1$. Finally, we need $a_2\ a_1 - a_0 > 0$ to show that all eigenvalues of the Jacobian $\mathbf{J}^*$ have a negative real part.

$$a_2\ a_1 - a_0 = \frac{4\left(25\lambda^6 + 294\lambda^4 - 208\lambda^3 + 1153\lambda^2 - 624\lambda + 1296\right)}{\left(\lambda^2+3\right)^2\left(3\lambda^2 - 8\lambda + 9\right)^2} = 4\frac{P_5(\lambda)}{P_1^2(\lambda)P_2^2(2)}$$

Following a similar procedure as above reveals that $P_5(\lambda)$ is always concave up and positive for any real transition bias. Thus, all Routh-Hurwitz conditions are satisfied, and the coexistence equilibrium is a linearly stable fixed point.

$N > 3$ phenotypes

We provide a Mathematica[4] script that computes the eigenvalues for phenotypes up to order of $N = 10$ and plots them for the range of the transition bias $\lambda \in [-1, 1]$. As an example, Supplementary Fig. 3 shows the eigenvalues for $N = 7$, which are all negative for realistic ranges of our model parameters. The script is available at DOI: 10.5281/zenodo.7294563. For a more rigorous argument we here provide a Lyapunov stability analysis. The system in Eq. 2 can be written more compactly in matrix form as

$$\dot{\boldsymbol{x}} = \left(1 - \frac{X}{K}\right)\boldsymbol{D}\boldsymbol{x} + \boldsymbol{A}\boldsymbol{x}, \tag{6}$$

where

$$\boldsymbol{D} = \begin{bmatrix} r_1 & & & \\ & r_2 & & \\ & & \ddots & \\ & & & r_N \end{bmatrix}, \tag{7}$$

and

$$\boldsymbol{A} = cr_1 \begin{bmatrix} -(1+\lambda) & 1-\lambda & & & & \\ 1+\lambda & -2 & 1-\lambda & & & \\ & 1+\lambda & -2 & 1-\lambda & & \\ & & \ddots & \ddots & \ddots & \\ & & & 1+\lambda & -2 & 1-\lambda \\ & & & & 1+\lambda & -(1-\lambda) \end{bmatrix}. \tag{8}$$

The matrix

$$\boldsymbol{B} = \left(1 - \frac{X}{K}\right)\boldsymbol{D} + \boldsymbol{A}, \tag{9}$$

allows us to write the system in Eq. 6 as $\dot{\boldsymbol{x}} = \boldsymbol{B}(t)\boldsymbol{x}$. Since all off-diagonal entries of $\boldsymbol{B}(t)$ are non-negative for $t \geq 0$, the system in Eq. 6 is positive (Theorem 3.1)[5] and $\boldsymbol{x}(t) \geq 0$ and $\boldsymbol{x}(t) \neq 0$ for all $t \geq 0$ meaning that, when the system starts with non-negative initial conditions, it stays non-negative throughout.

To study the dynamics of this system, define the scalar function V as

$$V = \frac{X}{K} - \ln\left(\frac{X}{K}\right) - 1, \tag{10}$$

and note that $V > 0$ for $X \neq K$ while $V = 0$ for $X = K$. Differentiating,

$$\dot{V} = \frac{X-K}{XK}\dot{X}, \tag{11}$$

where from Eq. 2, the definition of X, and telescoping,

$$\dot{X} = \sum_{j=1}^{N} \dot{x}_j = \left(1 - \frac{X}{K}\right)\sum_{j=1}^{N} r_j x_j, \tag{12}$$

so that we can write the right-hand side of Eq. 11 in closed form as

$$\dot{V} = -\frac{(X-K)^2}{XK^2}\sum_{j=1}^{N} r_j x_j. \tag{13}$$

This expression shows that the derivative of V is negative whenever $X \neq K$, while it vanishes at $X = K$. Therefore, V is a Lyapunov function for the dynamics of X and $X^* = K$ is a globally stable equilibrium of X. Notably, this stability result is independent of the dynamics of the system that originate from the second term on the right-hand side of Eq. 6.

Due to this result, the system in Eq. 6 eventually approaches the linear system

$$\dot{\boldsymbol{x}} = \boldsymbol{A}\boldsymbol{x}, \tag{14}$$

as $t \to \infty$, where $\boldsymbol{A}$ is time independent. The system of Eq. (2) has fixed points when the two terms, growth dynamics and transition dynamics, in Eq. (6) vanish simultaneously. All phenotype configurations satisfying $X = K$ make the first term vanish.

The transition matrix $\boldsymbol{A}$ can be seen as an irreducible generator of a continuous-time Markov chain. For $\lambda \in (-1, +1)$, the irreducibility follows from the fact that you can go from any phenotype i to any other phenotype $j \neq i$ through some path on the directed graph of $\boldsymbol{A}$. Then, by Lemma A.2 from Reference 7, 0 is an eigenvalue of $\boldsymbol{A}$ with multiplicity 1, and all other eigenvalues have negative real parts. For $\lambda \pm 1$, the multiplicity 1 of eigenvalue 0 follows from the diagonal of the resulting triangular matrix. Multiplying any vector that is parallel to the unique eigenvector $\boldsymbol{v}$, corresponding to the 0 eigenvalue, to the right side of $\boldsymbol{A}$ will yield 0. Therefore Eq. (1), obtained by scaling eigenvector $\boldsymbol{v}$ such that the components of $\boldsymbol{v}$ sum to K, is the unique solution to Eq. (6) for all N.

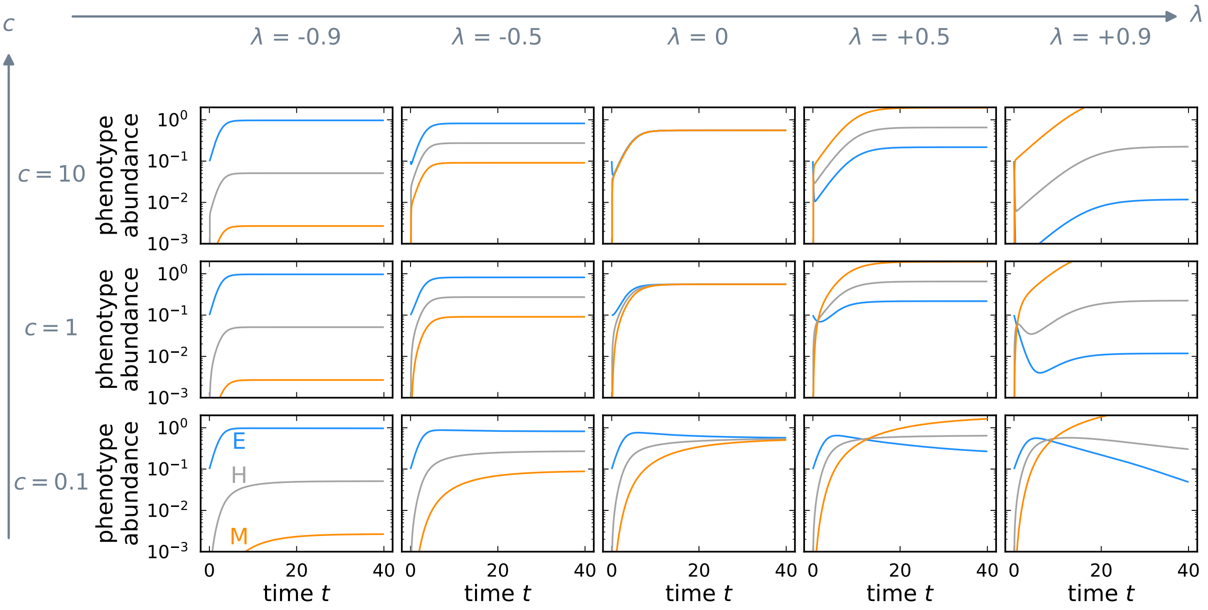

Supplementary Figure 4: Unequal competition allows tumor burdens higher than the carrying capacity. The panels show approach to the stable phenotype distribution from the same initial condition, $(x_1, x_2, x_3) = (\frac{1}{10}, 0, 0)$, for different combinations of transition speed c and transition bias λ. We assume that cells consume resources proportional to their growth rate, $\alpha_i = r_i \cdot (1 \; \textit{time unit})$. Since mesenchymal cells consume fewer resources, the higher the mesenchymal proportion, the more cells can be sustained above the carrying capacity.

Model extensions

Unequal competition

In the original model, we assumed that all phenotypes share the resources identically. However, resource utilization of phenotypes may differ and lead to a different competitiveness of phenotypes. To account for this aspect, here we extend the model by including phenotype-specific competition coefficients α_i. Thus, we replace X in Eqs. (2) with $\tilde{X} = \sum_{j=1}^{N} \alpha_j \, x_j$ and obtain for the untreated dynamics

$$
\begin{aligned}
\frac{dx_1}{dt} &= r_1 \, x_1 \left(1 - \frac{\tilde{X}}{K}\right) + c \, r_1 \left[(1-\lambda) \, x_2 - (1+\lambda) \, x_1)\right] \\
&\vdots \\
\frac{dx_i}{dt} &= r_i \, x_i \left(1 - \frac{\tilde{X}}{K}\right) + c \, r_1 \left[(1+\lambda) \, x_{i-1} + (1-\lambda) \, x_{i+1} - 2 \, x_i)\right] \\
&\vdots \\
\frac{dx_N}{dt} &= r_N \, x_N \left(1 - \frac{\tilde{X}}{K}\right) + c \, r_1 \left[(1+\lambda) \, x_{N-1} - (1-\lambda) \, x_N)\right]
\end{aligned}
\tag{15}
$$

This set of equations has a saddle point and a stable equilibrium similar to the original model. The state with no cells is an unstable equilibrium. The stable equilibrium is given

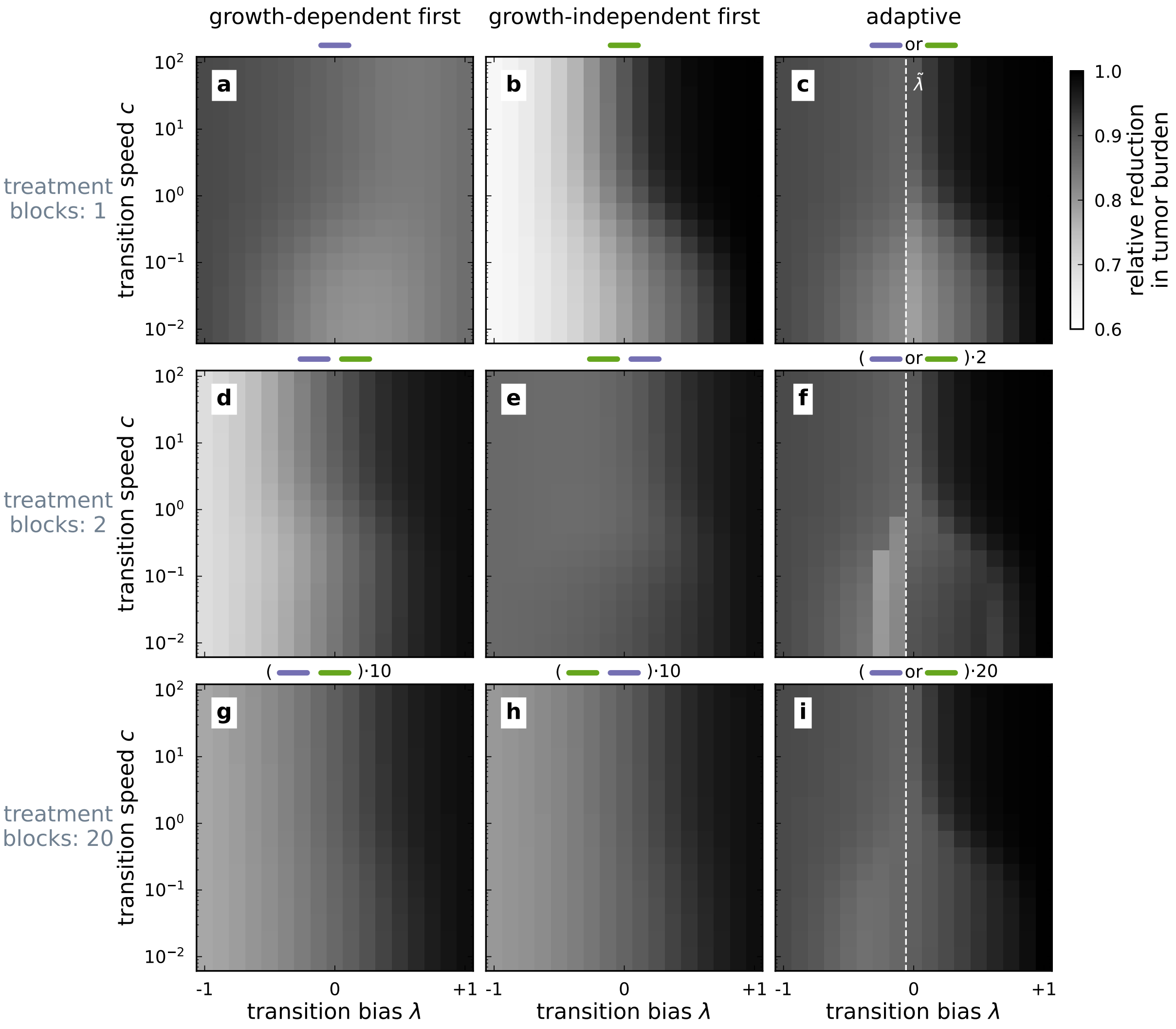

Supplementary Figure 5: Unequal competition improves treatment efficiencies for unbiased and mesenchymal-biased tumors. The heatmap shows the relative reduction in tumor burden from the abundance fixed point $\frac{\tilde{K}-X}{\tilde{K}}$ at the stable phenotype distribution for unequal competition where $\tilde{K} = \frac{K \sum_{j=1}^{3} (1-\lambda)^{3-j} (1+\lambda)^{j-1}}{\sum_{j=1}^{3} \alpha_j (1-\lambda)^{3-j} (1+\lambda)^{j-1}}$. The intensity of growth-independent treatment is $m_I \approx 0.65$. Darker colors represent a higher reduction and, thus, a better outcome. We evaluate the effect of splitting the treatment period into multiple treatment blocks (rows) and investigate different treatment schemes with either predefined or adaptive treatment sequences (columns).

by $\tilde{x}_i^* = K \frac{(1-\lambda)^{N-i} (1+\lambda)^{i-1}}{\sum_{j=1}^{N} \alpha_j (1-\lambda)^{N-j} (1+\lambda)^{j-1}}$. Note that the stable equilibrium is obtained without any assumptions on the competition coefficients and therefore is valid for any non-negative competition coefficients. The competition coefficients α_i affect only the carrying capacity in the form of a constant scaling factor $\kappa = \frac{\sum_{j=1}^{N} (1-\lambda)^{N-j} (1+\lambda)^{j-1}}{\sum_{j=1}^{N} \alpha_j (1-\lambda)^{N-j} (1+\lambda)^{j-1}}$. The stable phenotype distribution with unequal competition is, thus, the equilibrium abundances $\tilde{x}_i^*$ divided by this scaled carrying capacity κK, e.g. $\Pr(i \mid \lambda, N) = \frac{\tilde{x}_i^*}{\sum_{j=1}^{N} \tilde{x}_j^*} = \frac{x_i^*}{\kappa K}$. Remarkably, the transition bias λ solely determines the stable phenotype distribution also in the case of unequal competition

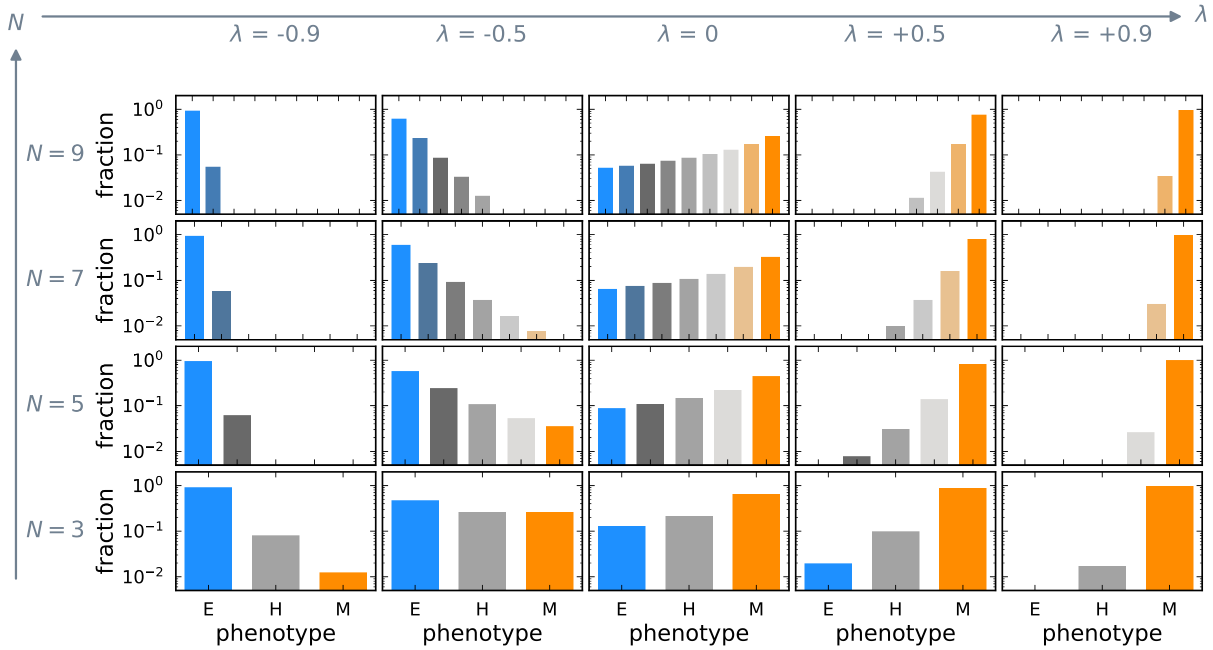

Supplementary Figure 6: Phenotype-dependent transition speed does not qualitatively affect the stable phenotype distribution. Each panel shows the equilibrium distribution of phenotypes for a fixed value of the transition bias and the number of phenotypes. The stable phenotype distribution changes with the transition bias λ. When there is no transition bias to switch to either an epithelial or a mesenchymal-like phenotype, i.e., $\lambda = 0$, the stable distribution is not uniform as faster proliferating cells transition faster. Transition bias towards the epithelial-like phenotype, $\lambda < 0$, leads to an increase in epithelial cells. Conversely, a transition bias towards mesenchymal-like phenotypes, $\lambda > 0$, leads to an increase in mesenchymal cells.

between phenotypes.

Notably, now the abundances, particularly of the mesenchymal phenotype, can exceed the carrying capacity K, i.e. $\kappa > 1$, up to an effective carrying capacity given by $\tilde{K} = \kappa K$ (Supplementary Fig. 4). Consider the case where mesenchymal-like cells use fewer resources than epithelial-like cells. Namely, we assume that the competition coefficients are proportional to the growth rate. Rescaling the competition coefficients then gives $\alpha_i = r_i \cdot 1$ (time unit) for the ith phenotype. Note that the units of growth rate and competition coefficients are different requiring the multiplication by 1 (time unit). This assumption incorporates a classical $r - K$ trade-off into our model with more proliferative but less competitive epithelial cells and less proliferative but more competitive mesenchymal cells[6]. This higher abundance of less proliferative but more competitive mesenchymal phenotypes increases the treatment efficiencies for intermediate and high transition biases (Supplementary Fig. 5). Similarly, when a phenotype is harmful to others $\alpha_j > 1$ the equilibrium abundance can be lower than the carrying capacity $\kappa < 1$.

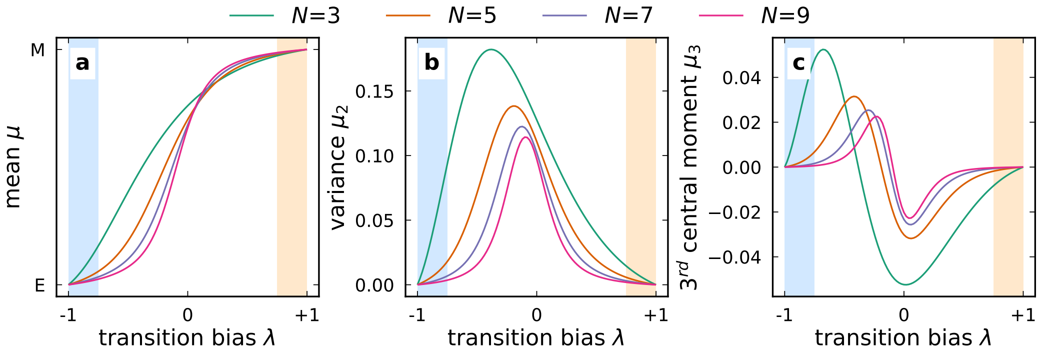

Supplementary Figure 7: Phenotype-dependent transition speed decreases the abundance of epithelial phenotypes. Plot specifics are identical to Supplementary Fig. 1. Including phenotype-dependent transition speed breaks the symmetry of the stable phenotype distribution. The overall mesenchymal proportion increases as fast-proliferating epithelial cells transition faster into mesenchymal cells. Thus, the growth-dependent treatment type loses efficiency due to a worse phenotype match for a tumor with phenotype-dependent transition speed.

Phenotype-dependent transition speed

Here, we investigate the effect of using $c\ r_i$ for the transition speed of phenotype i instead of $c\ r_1$, i.e., phenotype transitions are coupled to cell divisions, and the transition rates are scaled by the growth rate of each phenotype.

$$\begin{aligned}
\frac{dx_1}{dt} &= r_1\ x_1 \left(1 - \frac{X}{K}\right) + c\ \left[r_2\ (1-\lambda)\ x_2 - r_1\ (1+\lambda)\ x_1)\right] \\
&\vdots \\
\frac{dx_i}{dt} &= r_i\ x_i \left(1 - \frac{X}{K}\right) + c\ \left[r_{i-1}\ (1+\lambda)\ x_{i-1} + r_{i+1}\ (1-\lambda)\ x_{i+1} - 2\ r_i\ x_i)\right] \\
&\vdots \\
\frac{dx_N}{dt} &= r_N\ x_N \left(1 - \frac{X}{K}\right) + c\ \left[r_{N-1}\ (1+\lambda)\ x_{N-1} - r_N\ (1-\lambda)\ x_N)\right]
\end{aligned} \tag{16}$$

This set of equations has two equilibria similar to the original model. One equilibrium is a saddle-node equilibrium of extinction, and the other is a stable equilibrium with a fixed phenotype distribution. This stable equilibrium is given by $\hat{x}_i^* = K \frac{r_1}{r_i} \frac{(1-\lambda)^{N-i}\ (1+\lambda)^{i-1}}{\sum_{j=1}^{N} \frac{r_1}{r_j}\ (1-\lambda)^{N-j}\ (1+\lambda)^{j-1}}$. The model dynamics with phenotype-dependent transition speed are qualitatively similar to the original model. In this case, however, the stable phenotype distribution depends on the phenotype growth rate, increasing the fraction of more mesenchymal phenotypes (Supplementary Fig. 6). This leads to stable phenotype distributions with negative third central moment for a larger transition bias range (Supplementary Fig. 7) and an efficiency loss of purely growth-dependent treatment (Supplementary Fig. 8).

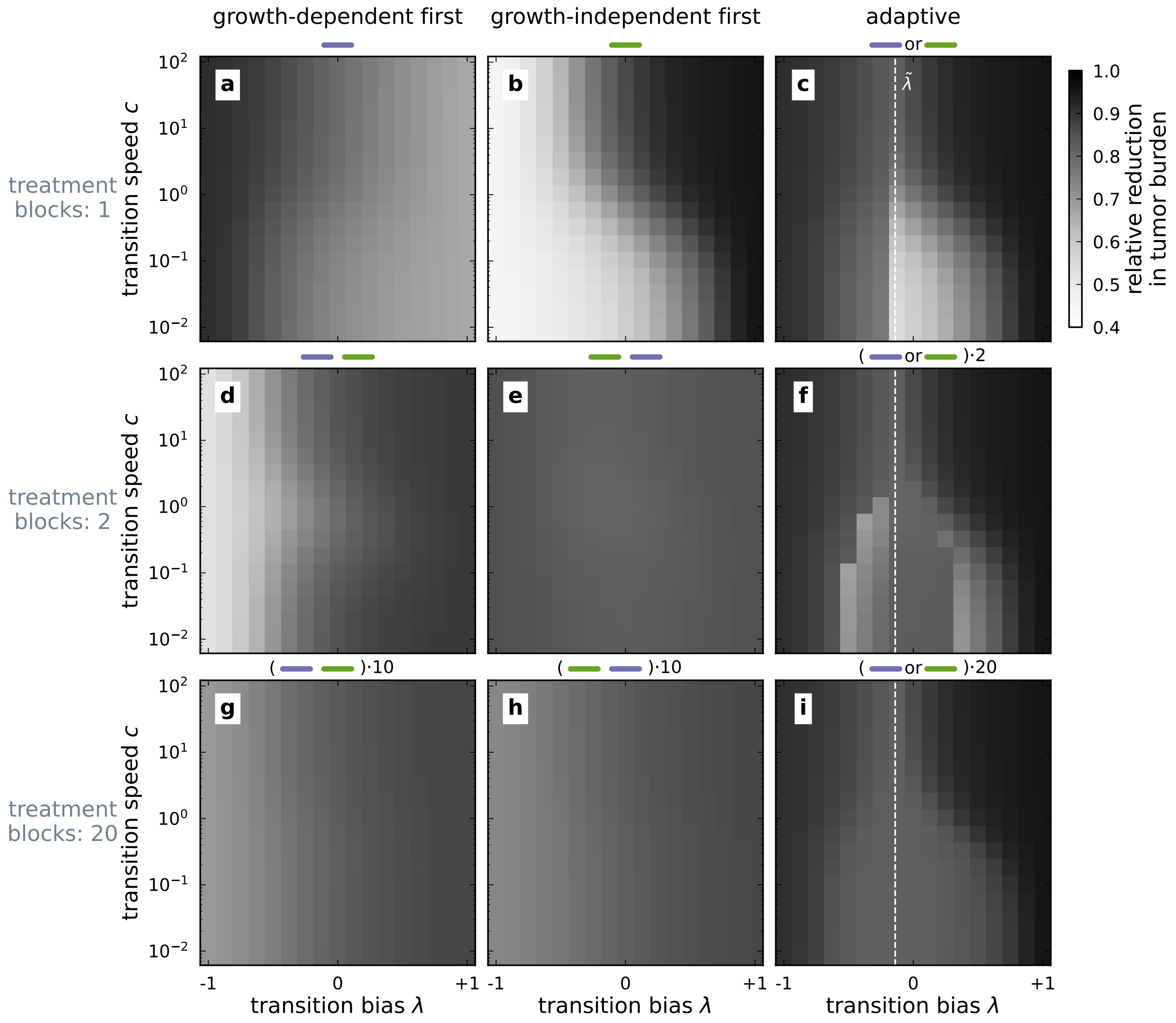

Supplementary Figure 8: Phenotype-dependent transition speed improves the treatment efficiency. The heatmap shows the relative reduction in tumor burden from the abundance fixed point $\frac{K-X}{K}$ at the stable phenotype distribution for phenotype-dependent transition speed. The intensity of growth-independent treatment is $m_I \approx 0.50$. Darker colors represent a higher reduction and, thus, a better outcome. We evaluate the effect of splitting the treatment period into multiple treatment blocks (rows) and investigate different treatment schemes with either predefined or adaptive treatment sequences (columns). The negative effect of transition speed is amplified by phenotype-dependent transition speed. Thus, the overall growth rate of the tumor is decreased, further reducing the tumor burden at the end of the treatment duration.

References

[1] Steven H. Strogatz. *Nonlinear Dynamics and Chaos*. CRC Press, 0 edition, May 2018. ISBN 978-0-429-96111-3.

[2] Shankar Sastry. *Nonlinear Systems*, volume 10 of *Interdisciplinary Applied Mathematics*. Springer New York, New York, NY, 1999. ISBN 978-1-4419-3132-0 978-1-4757-3108-8.

[3] Feliks R. Gantmacher. *The Theory of Matrices. Vol. 2*, volume 2. American Mathematical Soc, Providence, RI, reprinted edition, 2009. ISBN 978-0-8218-2664-5.

[4] Inc. Wolfram Research. Mathematica. Wolfram Research, Inc., 2021.

[5] D.S. Bernstein and S.P. Bhat. Nonnegativity, reducibility, and semistability of mass action kinetics. In *Proceedings of the 38th IEEE Conference on Decision and Control (Cat. No.99CH36304)*, volume 3, pages 2206–2211, Phoenix, AZ, USA, 1999. IEEE. ISBN 978-0-7803-5250-6.

[6] Eric R. Pianka. On r- and K-Selection. *The American Naturalist*, 104(940):592–597, 1970. ISSN 00030147, 15375323.

[7] George Yin and Qing Zhang. *Continuous Time Markov Chains and Applications: A Two-Time-Scale Approach.* Number 37 in Applications of Mathematics. Springer, New York Berlin Heidelberg, 2nd ed edition, 2013. ISBN 978-1-4899-9118-8 978-1-4614-4345-2.

[8] Saumil Shah, Lisa-Marie Philipp, Stefano Giaimo, Susanne Sebens, Arne Traulsen, and Michael Raatz. Understanding and leveraging phenotypic plasticity during metastasis formation - Dataset, May 2023.

[9] Saumil Shah, Lisa-Marie Philipp, Stefano Giaimo, Susanne Sebens, Arne Traulsen, and Michael Raatz. Understanding and leveraging phenotypic plasticity during metastasis formation - Code. Zenodo, May 2023.
